# Ultrasound-assisted enhancement of microbial, physicochemical, and bioactive properties in *Lactobacillus helveticus-*fermented milk

**DOI:** 10.1016/j.fochx.2025.102724

**Published:** 2025-07-02

**Authors:** Aliakbar Gholamhosseinpour, Seyed Mohammad Bagher Hashemi, Hamideh Khosravi Mazydi

**Affiliations:** a Department of Food Science and Technology, Faculty of Agriculture, Jahrom University, Jahrom, Iran; b Department of Food Science and Technology, Faculty of Agriculture, Fasa University, Fasa, Iran

**Keywords:** Fermented Milk, *Lactobacillus helveticus*, Ultrasound, Bioactive properties

## Abstract

This research assessed the impacts of ultrasound on fermentation dynamics and bioactive properties in milk fermented with *Lactobacillus helveticus*. Ultrasound was applied to milk (MU + L), bacterial culture (M + LU), both (MU + LU), or the inoculated mixture [(M + L)U], and samples were analysed during a 24 h fermentation. Results demonstrated that ultrasound significantly enhanced microbial viability, especially in MU + LU. Acidification occurred more rapidly in ultrasound-treated samples, with MU + LU achieving the lowest pH(3.31). Ultrasound also improved antioxidant potential, with DPPH radical scavenging and ascorbate oxidation inhibition reaching 79.8 % and 28.4 %, respectively, in MU + LU. Furthermore, inhibitory activities against α-amylase, α-glucosidase, ACE, and lipase were significantly higher in ultrasound-treated samples, again with MU + LU showing the most pronounced effects (47.2 %,43.4 %,42.9 %, and 34.6 %, respectively). These findings highlight the synergistic advantages of applying ultrasound to milk and culture, offering a promising green, non-thermal approach to accelerate fermentation and improve microbial viability, antioxidant activity, and functional health-promoting properties in fermented dairy products.

## Highlights

• Ultrasound-assisted fermentation markedly enhanced L. *helveticus* viability.
• Ultrasound application accelerated pH decline and fermentation kinetics.
• Combined ultrasound treatment improved the antioxidant activity of fermented milk.
• Pre-treatment of milk and culture boosted α-amylase and α-glucosidase inhibition.
• Ultrasound synergistically improved ACE and lipase inhibitory activities.

## 1 Introduction

With the increasing awareness of consumers regarding diet and health, the demand for functional foods is on the rise. Fermented dairy products hold a prominent position due to their diverse bioactive properties. Therefore, the application of processing techniques that preserve the nutritional value, quality, and bioactive properties of these products without compromising their integrity is of great significance (Kaveh et al., 2023). In this context, fermented milks have attracted considerable attention owing to their enhanced nutritional and therapeutic potential. Ensuring the quality of such dairy products plays a critical role within the broader framework of food safety regulations (Bondoc & Șindilar, 2002). However, recurring quality concerns often arise due to issues related to production practices, hygiene management, and storage conditions (Bondoc, 2007). To address these limitations and further improve the functional potential of fermented milk, novel non-thermal processing methods are being explored.

Fermented milk products are among the most popular dairy products, recognized for their health benefits and extended shelf life (Elfahri et al., 2016). These products are primarily produced by adding bacterial cultures to raw or heat-treated milk. The bacterial starters used in their production mainly belong to the genera *Lactobacillus*, *Lactococcus*, and *Streptococcus*, which contribute exceptional nutritional and flavor-enhancing properties (Gholamhosseinpour & Hashemi, 2019). The health-promoting potential of fermented milk has been well established, with evidence highlighting its roles in improving gastrointestinal function, regulating serum cholesterol and blood pressure, reducing the likelihood of certain cancers, enhancing lactose digestion, and contributing to overall metabolic health (Deepak et al., 2016; Shiby & Mishra, 2013).

Lactic acid bacteria (LAB) are a group of microorganisms widely used in food fermentation due to their distinctive properties. These bacteria improve organoleptic properties, enhance nutritional value, promote health, and increase food safety. Recognized as GRAS (Generally Recognized as Safe) by the U.S. Food and Drug Administration, LAB play a crucial role in the dairy industry, where they are employed in the production of a wide range of fermented dairy products (Shokri et al., 2020).

*Lactobacillus helveticus* is a Gram-positive, rod-shaped, thermophilic, and homofermentative lactic acid bacterium widely used in the production of fermented dairy products (Chelladhurai et al., 2023; Guan et al., 2021). This bacterium metabolizes lactose into lactic acid as its primary fermentation product and exhibits a high tolerance to acidic conditions (Chelladhurai et al., 2023). The fermentation of milk by *L. helveticus* significantly enhances the production of bioactive peptides, including antioxidant peptides, angiotensin-converting enzyme (ACE) inhibitors, as well as anti-cancer and anti-inflammatory peptides (Gao et al., 2022). Recent clinical evidence shows that consuming heat-treated L. *helveticus* CP790-fermented milk significantly improves gastrointestinal health in adults by reducing symptoms of constipation and modulating the composition of the gut microbiota (Tanihiro et al., 2024). This effect is believed to be partly due to the presence of bioactive compounds generated during fermentation, which function as postbiotics and support gut health. Moreover, a comprehensive recent review identifies L. *helveticus* as a major producer of various bioactive milk peptides with antioxidant, angiotensin-converting enzyme (ACE) inhibitory, anti-inflammatory, and other health-promoting properties (Nielsen et al., 2024). These findings further reinforce the key role of L. *helveticus* in functional fermented dairy products that offer potential health benefits. Due to its vigorous proteolytic activity, *L. helveticus* strains are commonly employed as starter cultures in the production of fermented milk and various types of cheese (Kilpi et al., 2007).

Ultrasound has gained considerable attention as an emerging non-thermal processing technology in the food sector, owing to its ability to reduce processing intensity while preserving nutritional and sensory properties, enhancing product quality, and improving microbial safety. In recent decades, its application in the food sector has expanded due to its high processing speed, non-destructive nature, cost-effectiveness, and ease of automation. This technology is widely utilized for various purposes, including drying, mixing, emulsification, degassing, extraction of bioactive compounds, crystallization of sugars and fats, and stimulation of living cells (Gholamhosseinpour & Hashemi, 2019; Majid et al., 2015; Ojha et al., 2018). Studies have shown that ultrasound-assisted dairy processing, particularly in fermented products, has attracted significant research interest. The most notable reported effects of ultrasound in the dairy industry include microbial inactivation, stimulation of cell growth, reduced fermentation time, accelerated substrate transport, improved acidification kinetics, equipment cleaning, increased membrane permeability, and enhanced homogenization efficiency (Gholamhosseinpour et al., 2020; Nguyen et al., 2009).

Ultrasound technology, as an emerging non-thermal processing method, has garnered considerable attention for its ability to enhance food processing efficiency and improve the techno-functional properties of dairy products. Numerous studies have examined the effects of ultrasound on the physicochemical, microbiological, and functional attributes of dairy products. However, to the best of our knowledge, no research has specifically investigated the application of ultrasound-treated L. *helveticus* in fermented milk production. This study investigated the effect of ultrasound treatment on L. *helveticus* and its role in the milk fermentation process. The microbial characteristics, antioxidant capacity, and bioactive properties of the final product were evaluated to determine whether this technology could enhance the functionality of L. *helveticus* during fermentation. The findings of this study provide valuable insights into the effects of ultrasound on milk fermentation and contribute to the creation of novel strategies to improve the production and quality of fermented dairy products.

## 2 Materials and methods

### 2.1 Microbial strain

*Lactobacillus helveticus* PTCC 1332 was obtained from the Food Hygiene Department of Fasa University of Medical Sciences and then activated in MRS broth at 37 °C for 24 h.

### 2.2 Sample preparation

UHT milk was purchased from Pegah Company (Fars province; Iran) and inoculated with L. *helveticus* (∼4.33 log CFU/mL) to prepare fermented milk. In order to investigate the effect of ultrasound, an ultrasonic bath (Elmasonic S 300H; 37 KHz, 300 W; Germany) was used according to the procedure of Gholamhosseinpour et al. (2024) with slight modifications. The ultrasound duration for all samples was 150 s at 30 °C. For this purpose, 50 mL of milk or bacterial culture medium was poured into a 100 mL beaker and placed in an ultrasonic bath. Five samples were used in this study. Bacteria were sonicated and added to milk (M + LU) in one sample. In another sample, milk without bacteria was sonicated and then bacteria were added to it (MU + L). In the subsequent sample, bacteria and milk were sonicated separately before combining the sonicated bacteria with the sonicated milk (MU + LU). In another sample, bacteria were added to milk and then the ultrasound process was performed [(M + L)U], and in the control sample (M + L), neither milk nor bacteria were sonicated. The fermentation process of the samples was performed for 24 h at 37 °C, and microbial and physicochemical tests were performed at intervals of every 8 h.

### 2.3 Microbial enumeration

MRS culture medium was used to count bacteria. For this purpose, 1 mL of the diluted samples was poured into a plate containing the culture medium after dilution with saline. The plates were placed at 37 °C for 48 h, and then the colonies were counted (Hashemi & Gholamhosseinpour, 2020b).

### 2.4 pH measurement

The pH of the fermented milk samples was measured at ambient temperature using a benchtop pH meter (Starter 2100F, OHAUS, USA), that had been properly calibrated prior to use.

### 2.5 DPPH radical scavenging activity

Radical scavenging activity was performed using the procedure of Pyo et al. (2005) with some modifications. The samples were mixed with 1 mL of methanol and then centrifuged (3000 *g*; 15 min). Then, the obtained supernatant was mixed with 1 mL of DPPH (0.1 mmol/L) and 1.5 mL of methanol. After 30 min, the absorbance was measured at 517 nm.

### 2.6 Inhibition of ascorbate autoxidation

A 0.1 mL of milk sample was mixed with 0.1 mL of ascorbate solution and 9.8 mL of phosphate buffer. After keeping the prepared mixture at 37 °C for 10 min, the absorbance was measured at 265 nm (Wang et al., 2006).

### 2.7 α-Amylase inhibition assay

Initially, the samples were adjusted to pH = 4 and then centrifuged (3000 *g*; 10 min). The resulting supernatant was filtered (0.22 μm), and 100 μL of it was mixed with 100 μL of α-amylase and kept at 37 °C for 5 min. Starch (1 %) was used to start the reaction, and 3,5-dinitrosalicylic acid (1 %) and sodium potassium tartrate (12 %) in 0.4 mol/L NaOH were used to end the reaction. Finally, the absorbance was read at 540 nm (Ayyash et al., 2018; Kim et al., 2004).

### 2.8 α-Glucosidase inhibition assay

To perform this test, 50 μL of the supernatant obtained according to the α-amylase test was combined with α-glucosidase (1 unit/mL) and kept for 10 min at 37 °C. To start the reaction, 50 μL of *p*-nitrophenyl α-D-glucopyranoside was used at 30 °C for 30 min, and 1 mL of Na_2_CO_3_ was used to finish the reaction. At the end, the absorbance was read at 400 nm (Ayyash et al., 2018; Kim et al., 2004).

### 2.9 Angiotensin I-converting enzyme (ACE) inhibitory activity

Briefly, to measure ACE inhibition activity, the hippuric acid was diluted in deionized water, and then the absorbance of the resulting solution was measured at 228 nm, and finally, the percentage of ACE inhibition was obtained (Cushman & Cheung, 1971; Hati et al., 2015).

### 2.10 Lipase inhibition activity

Lipase inhibition activity of fermented samples was measured over 24 h using the Gil-Rodríguez and Beresford (2019), Humbert et al. (2006) and Sergent et al. (2012) methods. Briefly, 0.5 mL of the sample was mixed with 2 mL of Tris-HCl buffer, followed by the addition of 50 μL of a 5 mmol/L solution of 4-nitrophenyl octanoate in dimethyl sulfoxide. The reaction was initiated by the addition of lipase enzyme, and after several incubation steps at 37 °C, the absorbance was read at 412 nm.

### 2.11 Statistical analysis

Statistical analysis was performed using one-way ANOVA to identify significant differences between groups, with Duncan's test applied for mean comparisons at a 5 % significance level. All statistical procedures were conducted using SPSS software (version 20.0, SPSS Inc., Chicago, IL, USA), while graphical representations of the data were generated using Microsoft Excel 2016.

## 3 Results and discussion

### 3.1 The viability of L. *helveticus* in fermented milk during fermentation

**Fig. 1:**
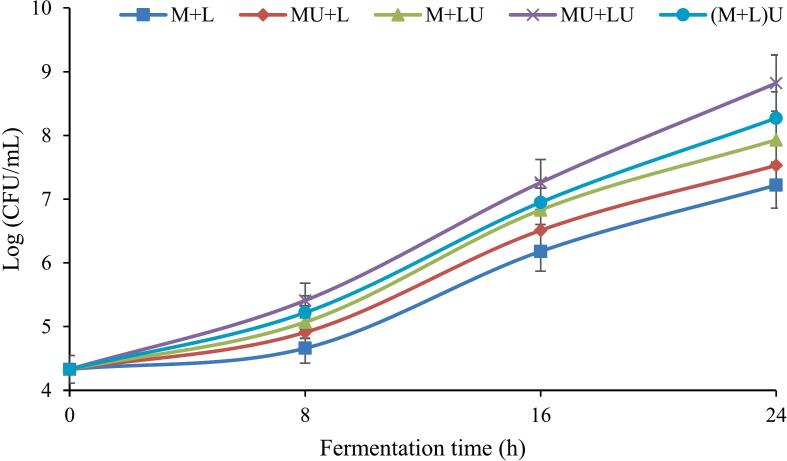
The effect of ultrasound treatment on the viability of L. *helveticus* in fermented milk during fermentation. (MU + L: milk ultrasonicated, M + LU: bacteria ultrasonicated, MU + LU: milk and bacteria ultrasonicated separately, (M + L)U: mixture ultrasonicated after inoculation, control: no ultrasound).

Fig. 1 illustrates the effect of ultrasound treatment and fermentation time on the population dynamics of L. *helveticus* in milk over 24 h. The results indicated that increasing fermentation time significantly (*P* ≤ 0.05) enhanced bacterial population growth across all treatments, with the highest L. *helveticus* count recorded at the end of fermentation. A slight increase in bacterial cell count was observed during the initial phase (0–8 h). This can be attributed to the lag phase, where bacteria must adapt to the environment and synthesize essential enzymes. In this phase, ultrasound-treated samples showed a slight increase compared to the control, likely due to improved nutrient accessibility induced by ultrasonic cavitation (Carrillo-Lopez et al., 2021). Bacterial growth accelerated significantly between 8 and 16 h (P ≤ 0.05), with ultrasound-treated samples demonstrating the highest growth rates. In the final stage (16–24 h), bacterial growth continued in all treatments. However, the rate of population increase declined, likely due to the accumulation of acidic metabolites, nutrient depletion, and pH changes limiting bacterial growth. Ultrasound treatment led to an overall increase in bacterial count, with the MU + LU treatment being the most effective, showing a 103.7 % increase, where the bacterial population rose from 4.33 log CFU/ml to 8.82 log CFU/ml after 24 h. The (M + L)U, M + LU, and MU + L treatments ranked next, with increases of 90.9 %, 83.1 %, and 73.9 %, respectively, while the control (M + L) exhibited the lowest increase (66.7 %).

The results of this study were consistent with the findings of other researchers who have reported the positive effects of ultrasound on Lactobacillus growth (Bolívar-Jacobo et al., 2023; Gammoh et al., 2020; Huang et al., 2019; Shokri et al., 2020). Low-frequency ultrasound enhances cell membrane permeability by creating transient pores, facilitating the transport of substances across the membrane. This process promotes the release of intracellular metabolites, such as enzymes, and stimulates metabolic activities, ultimately supporting bacterial growth and proliferation (Huang et al., 2017; Mota et al., 2018). Ultrasound treatment also stimulates lactose uptake and the secretion of intracellular enzymes like β-galactosidase, further facilitating metabolic processes and contributing to bacterial growth (Carrillo-Lopez et al., 2021; Gholamhosseinpour & Hashemi, 2019). Additionally, ultrasound increases the transfer of oxygen and nutrients into cells, induces degassing, and creates anaerobic conditions, all enhancing LAB activation. Moreover, the elimination of cellular waste and the production of cell lysates induced by ultrasound play a crucial role in enhancing the growth of LAB (Akdeniz & Akalın, 2022; Hashemi & Gholamhosseinpour, 2020b; Yeo & Liong, 2013).

### 3.2 Changes in pH of fermented milk during fermentation

**Fig. 2:**
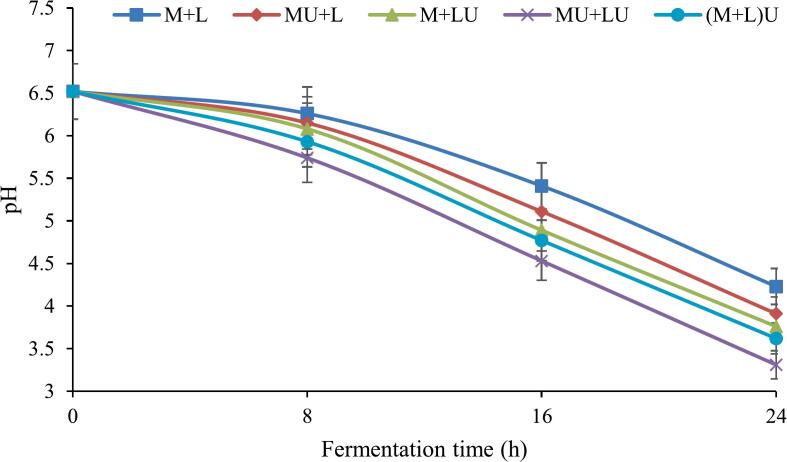
The effect of ultrasound treatment on the pH of fermented milk during fermentation. (MU + L: milk ultrasonicated, M + LU: bacteria ultrasonicated, MU + LU: milk and bacteria ultrasonicated separately, (M + L)U: mixture ultrasonicated after inoculation, control: no ultrasound).

Fig. 2 illustrates the pH changes of various samples over the 24 h fermentation period. A decrease in pH was observed across all treatments during the 24 h fermentation period, indicating the production of organic acids, primarily lactic acid, due to the metabolic activity of L. *helveticus*. The most significant pH decline occurred between 16 and 24 h in all treatments, with reductions ranging from 21.8 % to 26.9 %, highlighting the peak phase of acid production during fermentation. In the control sample (M + L), the pH decreased from 6.52 to 4.23. In treatments where ultrasound was applied to milk (MU + L) or bacteria (M + LU), the pH dropped more rapidly, reaching 3.91 and 3.76, respectively, by the end of fermentation. This suggests that ultrasound enhances fermentation kinetics by modifying milk structure or altering bacterial membrane permeability, thereby facilitating increased acid production. The lowest pH (3.31) was observed in the MU + LU treatment, indicating a synergistic effect that significantly accelerated fermentation and acid synthesis. In the (M + L)U treatment, the pH decline was more pronounced than in the control but less than in MU + LU (pH 3.62). This suggests that while ultrasound treatment of the combined system influenced fermentation kinetics, its impact was less pronounced than when applied separately to milk and bacteria.

Lactic acid bacteria utilize lactose as a primary carbon source, metabolizing it into lactic acid, thereby lowering the pH (Shabbir et al., 2023). In the course of lactic fermentation, lactose undergoes intracellular breakdown by β-galactosidase, producing glucose and galactose, which are then converted into lactic acid through further metabolic processes (Gholamhosseinpour et al., 2024). Ultrasonication promotes the release of β-galactosidase from LAB cells. The released enzyme enhances the availability of glucose and galactose for LAB, leading to increased lactic acid production and a further acceleration of the acidification process (Nguyen et al., 2009).

It was reported that ultrasonication facilitated lactose hydrolysis in milk in the presence of a starter culture (*L. delbrueckii*) (Ashokkumar et al., 2010). Similarly, Düven et al. (2021) observed that ultrasonication accelerated the growth of Lactobacillus and Lactococcus in kefir, leading to increased lactic acid production and a more pronounced pH reduction compared to non-sonicated samples. Applying ultrasound to LAB disrupts the cell wall structure, enhancing membrane permeability, improving substrate transport, and increasing metabolite production, ultimately accelerating fermentation (Hashemi & Gholamhosseinpour, 2020a; Patist & Bates, 2008). These effects correlate with a significant decrease in pH. The simultaneous ultrasonication of milk and bacterial cells further enhances lactic acid production by increasing substrate accessibility and stimulating microbial activity, suggesting a synergistic effect between ultrasound treatment and fermentation kinetics.

### 3.3 The antioxidant activities of fermented milk during fermentation

**Fig. 3:**
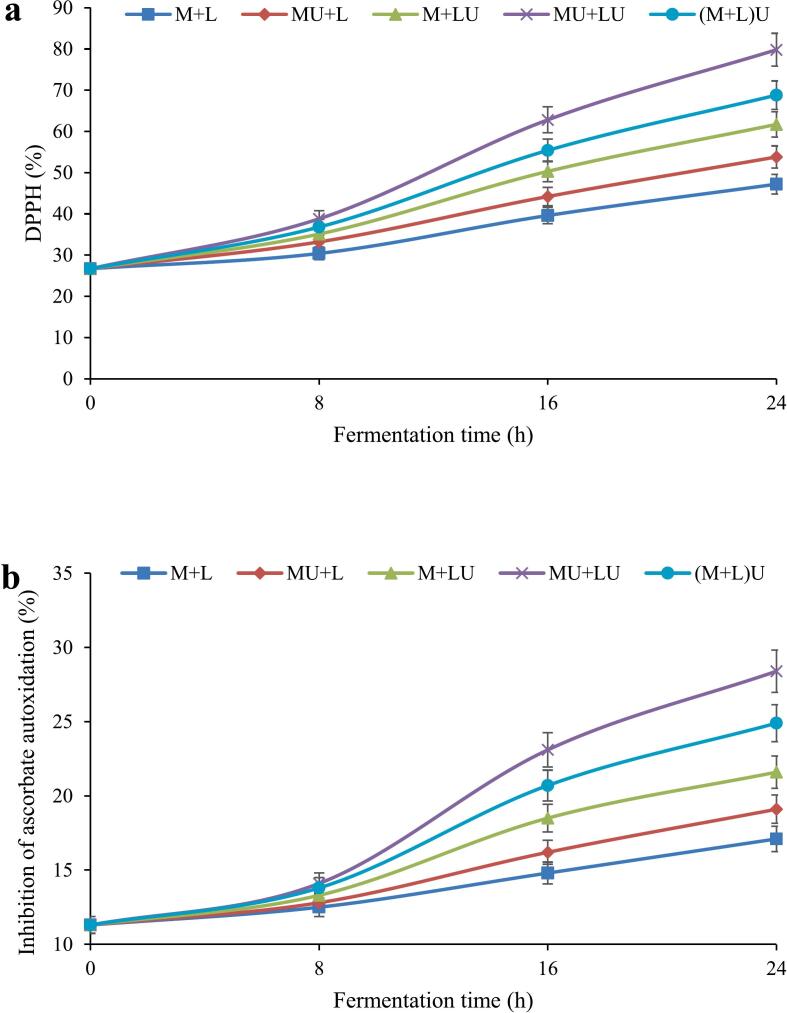
The effect of ultrasound treatment on antioxidant (DPPH (a) and ascorbate oxidation inhibition (b)) activities of fermented milk during fermentation. (MU + L: milk ultrasonicated, M + LU: bacteria ultrasonicated, MU + LU: milk and bacteria ultrasonicated separately, (M + L)U: mixture ultrasonicated after inoculation, control: no ultrasound).

Fig. 3a displays the changes in the radical scavenging activity of the fermented milk samples subjected to ultrasound, measured via the DPPH assay. A significant enhancement (*P* ≤ 0.05) in antioxidant potential was observed across all treatments throughout the 24 h fermentation process, although the magnitude of improvement differed depending on the ultrasound treatment. The highest enhancement in antioxidant activity was observed between 8 and 16 h of fermentation, corresponding to the peak of microbial activity and the production of bioactive compounds. Among the treatments, MU + LU exhibited the highest antioxidant capacity, increasing from 26.7 % at time zero to 79.8 % at 24 h. In contrast, the control sample (M + L) displayed the lowest antioxidant capacity, reaching 47.2 % by the end of fermentation. Ultrasound treatment of milk (MU + L) resulted in a higher antioxidant capacity than the control (53.8 %), whereas ultrasonication of bacteria (M + LU) had a greater effect, increasing the antioxidant capacity to 61.7 %. The (M + L)U treatment demonstrated a higher antioxidant capacity than MU + L and M + LU, but remained lower than MU + LU (68.8 % at 24 h), indicating that separate sonication of milk and bacteria had a more pronounced impact on enhancing the antioxidant potential of the final product. Overall, fermentation alone contributed to increased antioxidant capacity; however, ultrasound treatment, particularly when applied to milk and bacteria simultaneously, exhibited a synergistic effect, leading to a final product with superior antioxidant properties. The concurrent sonication of both milk and bacterial culture (MU + LU) appears to synergistically optimize substrate structure and enzymatic efficiency, thereby maximizing the release of antioxidative compounds. The pronounced enhancement observed between 8 and 16 h of fermentation corresponds to peak microbial metabolism and proteolytic activity.

Ultrasound-assisted modification of ascorbate oxidation inhibition in various fermented milk samples is depicted in Fig. 3b. As can be seen, ascorbate oxidation inhibition significantly (*P* ≤ 0.05) increased in all treatments over time, although the extent of this increase varied among treatments. The most substantial enhancement occurred between 8 and 16 h of fermentation, likely due to increased metabolic activity and the release of antioxidant compounds. Among the treatments, MU + LU exhibited the highest oxidation inhibition capacity, increasing from 11.3 % at the beginning to 28.4 % after 24 h, demonstrating a significantly greater (P ≤ 0.05) inhibitory capacity than the other treatments. In contrast, the control sample (M + L) showed the lowest increase, reaching 17.1 %. The MU + L treatment improved antioxidant properties compared to the control (19.1 %), whereas ultrasound treatment of bacteria (M + LU) had a greater effect, increasing oxidation inhibition to 21.6 %. Additionally, the (M + L)U treatment demonstrated a higher ascorbate oxidation inhibition capacity than all treatments except MU + LU, indicating that the simultaneous sonication of milk and bacteria substantially enhanced ascorbate oxidation inhibition.

Researchers have reported an increase in antioxidant activity during the fermentation of milk. This enhancement is primarily attributed to the presence of protein peptides, cell lysis products, extracellular metabolites, and hydrolyzed milk components (Balakrishnan & Agrawal, 2014; Begunova et al., 2020; Uluko et al., 2015). This enhancement is not merely a byproduct of fermentation, but is deeply rooted in the biochemical transformations that occur during this process.

Proteolytic enzymes of LAB appear to be the key factors responsible for releasing antioxidant peptides, particularly those with specific amino acid sequences like hydrophobic, aromatic, or sulfur-containing residues that have strong radical scavenging properties. The increased concentration of these peptides and free amino acids leads to an overall improvement in the antioxidant properties of the product (Gholamhosseinpour & Hashemi, 2019). The production of antioxidant peptides is highly strain-dependent (Begunova et al., 2020; Hashemi & Gholamhosseinpour, 2020a). It has been well established that L. *helveticus* possesses one of LAB's most complex proteolytic systems. This complexity enables the breakdown of casein into a diverse array of bioactive peptides with antioxidant properties. In almost all the LAB strains, a cell envelope proteinase (CEP) is present in the cell wall, playing a crucial role in protein degradation and peptide release. However, some studies have reported that most strains of L. *helveticus* contain more than one CEP, making this species one of the most potent proteolytic starters among LAB (Namdari & Nejati, 2016).

Gholamhosseinpour and Hashemi (2019) reported that pre-fermentation ultrasound treatment improved fermented milk's DPPH radical scavenging potential compared to untreated samples. Ultrasound processing likely enhances these proteolytic processes by promoting cell membrane permeability and protein denaturation, thereby facilitating the access of enzymes to substrate molecules.

Ultrasound-assisted milk processing has been shown to promote the release of peptides with antioxidant potential by mechanisms involving cavitation, microstreaming, and mechanical disruption of protein structures, which may expose hydrophobic regions and increase enzymatic accessibility. This leads to significantly greater radical scavenging activity in treated samples than untreated ones (Uluko et al., 2015). Potoroko et al. (2018) also observed increased antioxidant activity in ultrasonicated fermented beverages, attributing this enhancement to the characteristics of raw milk, protein dispersion level, lactose content, and fat fraction. Additionally, Munir et al. (2020) attributed the increased proteolysis and antioxidant activity observed in pre-sonicated cheese milk to cavitation-induced structural modifications in proteins, such as partial unfolding and exposure of reactive amino acid residues. Finally, Ma'mon et al. (2018) stated that increasing the ultrasound amplitude enhances antioxidant activity by partially disrupting intermolecular and intramolecular hydrogen bonds in β-lactoglobulin, thereby increasing its reactivity and interaction with free radicals.

Thus, the combination of enhanced proteolysis, structural modifications in milk proteins, and microbial stimulation explains the significantly greater antioxidant capacity in the MU + LU treatment compared to others. The superior DPPH scavenging and ascorbate oxidation inhibition observed in MU + LU samples suggest that this non-thermal, energy-efficient strategy can be effectively employed to elevate the functional and health-promoting potential of fermented dairy products.

### 3.4 α- amylase and α-glucosidase inhibitory activities of fermented milk during fermentation

**Fig. 4:**
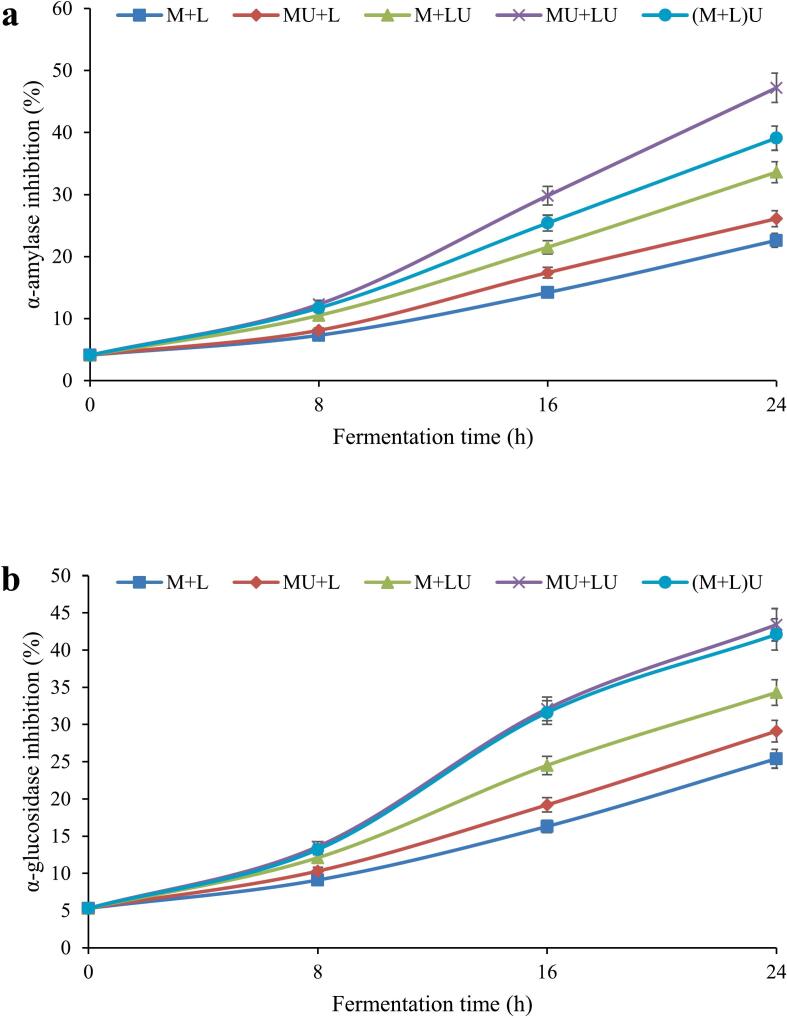
The effect of ultrasound treatment on α-amylase (a) and α-glucosidase (b) inhibition of fermented milk during fermentation. (MU + L: milk ultrasonicated, M + LU: bacteria ultrasonicated, MU + LU: milk and bacteria ultrasonicated separately, (M + L)U: mixture ultrasonicated after inoculation, control: no ultrasound).

Fig. 4a illustrates the effect of ultrasound on α-amylase inhibition in fermented milk throughout the fermentation period. The results demonstrated a significant (*P* ≤ 0.05) increase in α-amylase inhibition across all treatments as fermentation time progressed, confirming the positive influence of the fermentation process on enzyme inhibition. In the control treatment (M + L), the lowest level of α-amylase inhibition was observed, increasing from 4.1 % at the start to 22.6 % at the end of fermentation. The MU + L treatment, compared to the control, showed a higher level of inhibition (26.1 % at 24 h), suggesting that ultrasound application during fermentation enhanced enzyme inhibition. The M + LU treatment exhibited an even greater increase in enzyme inhibition than the other treatments (33.6 % at 24 h). Ultrasound treatment applied concurrently to milk and bacterial culture (MU + LU) resulted in the highest α-amylase inhibition (47.2 %) after 24 h of fermentation, demonstrating the most significant effect (P ≤ 0.05) among all tested modalities. Furthermore, the (M + L)U treatment also demonstrated substantial inhibition (39.1 % at 24 h), which was lower than that of MU + LU but higher than the other treatments.

The inhibitory potential of α-glucosidase under various ultrasound treatments is depicted in Fig. 4b. As can be observed, α-glucosidase inhibition significantly (P ≤ 0.05) increased over time across all treatments, with the magnitude of this increase being influenced by ultrasound treatment. In the control sample (M + L), enzyme inhibition rose from 5.3 % at the beginning of fermentation to 25.4 % at the end, indicating the effect of fermentation in producing compounds that inhibit this enzyme. Overall, treatments subjected to ultrasound exhibited higher inhibition of α-glucosidase. Compared to the control, the MU + L treatment demonstrated greater enzyme inhibition at each time point, reaching 29.1 % at 24 h. The M + LU treatment showed an even higher inhibition of 34.3 %, surpassing that of MU + L. This suggests that ultrasound has a more pronounced effect on L. *helveticus* than on milk, potentially producing more bioactive metabolites. The MU + LU treatment, where both components (milk and bacteria) were subjected to ultrasound, exhibited the highest inhibition at all times, reaching 43.4 % at 24 h, suggesting a synergistic effect of ultrasound in enhancing anti-diabetic compounds. Additionally, the (M + L)U treatment recorded a 42.1 % inhibition at the end of fermentation. Although α-glucosidase inhibition in the (M + L)U treatment was lower than in MU + LU during fermentation, this difference was insignificant.

Type 2 diabetes is a significant global health issue and is the ninth leading cause of death worldwide. A conventional therapeutic strategy for managing type 2 diabetes involves slowing carbohydrate digestion and reducing postprandial hyperglycemia by inhibiting enzymes like α-amylase and α-glucosidase (Ben-Miled et al., 2023). Previous studies have highlighted the superior ability of fermented dairy products to inhibit α-amylase and α-glucosidase enzymes compared to their unfermented counterparts (Ayyash et al., 2018; Hashemi & Gholamhosseinpour, 2020a; Shukla et al., 2023). This increased efficacy is primarily attributed to the release of bioactive peptides from intact proteins, which occurs through the action of bacterial proteases during fermentation. These peptides are directly responsible for the observed enzyme inhibition in fermented dairy products (Mudgil et al., 2024). One possible mechanism of inhibition involves the interaction of peptides with the enzyme's active site through hydrophobic bonds, although the exact inhibitory mechanism remains to be fully elucidated. Additionally, the involvement of hydrophobic amino acids in the inhibitory effects of peptides on α-glucosidase and α-amylase has been confirmed (Al-Dhaheri et al., 2017). Evidence also suggests that extracellular polysaccharides (EPS) secreted by these bacteria exhibit bioactivity and contribute to the inhibition of both enzymes (Al-Dhaheri et al., 2017; Zhang et al., 2023).

Numerous investigations have demonstrated that ultrasound treatment can enhance the inhibitory activity against α-glucosidase and α-amylase enzymes (Gholamhosseinpour et al., 2024; Hashemi & Gholamhosseinpour, 2020a; Kwiatkowska et al., 2011; Yu et al., 2014). Ultrasound-mediated inhibition of α-amylase and α-glucosidase occurs via multiple mechanisms. The application of ultrasound generates mechanical forces, including microjets and shock waves induced by the collapse of cavitation bubbles, which disrupt the cell walls of LAB and facilitate the release of intracellular proteases. These proteases hydrolyze proteins, producing bioactive peptides that inhibit α-amylase and α-glucosidase activity (Gholamhosseinpour et al., 2024). Moreover, ultrasound treatment can enhance the activity of the released intracellular enzymes, thereby promoting the generation of inhibitory peptides (Kwiatkowska et al., 2011). Additionally, ultrasound has been shown to stimulate the biosynthesis of EPS by LAB, further enhancing the inhibition of these enzymatic activities (Zhang et al., 2023). Ultrasound pretreatment facilitates the release of bioactive peptides by enhancing enzymatic accessibility to peptide bonds. This effect is attributed to acoustic cavitation, which reduces the size of fat globules, typically surrounded by casein micelles and whey proteins, thereby increasing the surface area available for enzyme-substrate interactions and improving proteolytic efficiency (Murtaza, Irfan, Hafiz, Ranjha, Rahaman, Murtaza et al., 2022). Structural modifications in milk casein induced by ultrasound include altering proteins' secondary and tertiary structures, disrupting hydrogen bonds and hydrophobic interactions, leading to molecular unfolding and/or protein fragmentation. These structural changes expose sulfhydryl and hydrophobic groups on the substrate, facilitating enzyme access to hydrolysis-prone regions of the polypeptide chain, ultimately improving the kinetic parameters of proteolysis under ultrasound treatment (Magalhães et al., 2022).

### 3.5 ACE inhibitory activity of fermented milk during fermentation

**Fig. 5:**
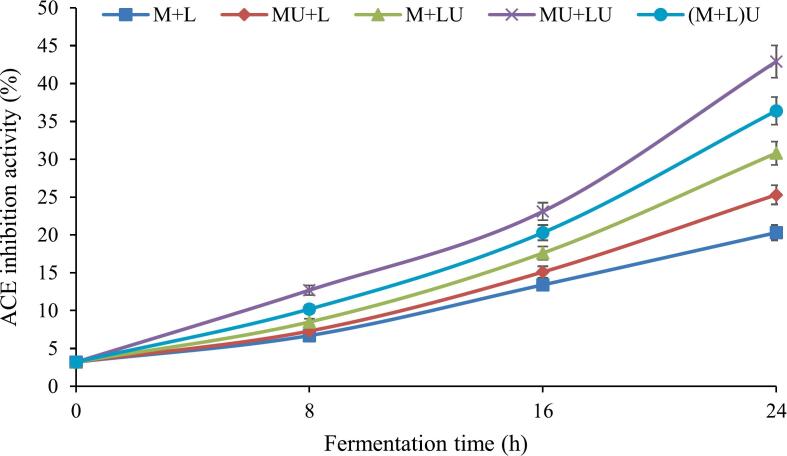
The effect of ultrasound treatment on ACE inhibitory activity of fermented milk during fermentation. (MU + L: milk ultrasonicated, M + LU: bacteria ultrasonicated, MU + LU: milk and bacteria ultrasonicated separately, (M + L)U: mixture ultrasonicated after inoculation, control: no ultrasound).

Fig. 5 illustrates the effect of ultrasonication on ACE inhibition in fermented milk throughout fermentation. The results indicated a significant (*P* ≤ 0.05) increase in ACE inhibition across all treatments as fermentation progressed. The control (M + L) exhibited the lowest ACE inhibition (20.3 % at 24 h). Ultrasound treatment applied either to milk (MU + L) or L. *helveticus* (M + LU) separately led to a greater increase in ACE inhibition compared to the control, reaching 25.3 % in MU + L and 30.8 % in M + LU by the end of fermentation. The highest ACE inhibition was observed in treatments where both milk and bacteria were subjected to ultrasound (MU + LU) or when the final mixture was ultrasonicated after blending [(M + L)U], achieving inhibition rates of 42.9 % and 36.4 %, respectively. These findings suggest that the simultaneous ultrasonication of milk and bacteria exerts a synergistic effect, enhancing biological activity and promoting the production of ACE-inhibitory peptides.

Milk serves as a rich source of peptides with bioactive properties, and those exhibiting ACE-inhibitory activity are commonly released during enzymatic hydrolysis of milk proteins throughout processes such as fermentation, gastrointestinal digestion, or food processing (Chen, Li, Xue, Kwok, Yang, Zhang, et al., 2015; Parmar et al., 2020). *L. helveticus* is known for producing various intracellular proteolytic enzymes, including cell-envelope proteinases, endopeptidases, aminopeptidases, and X-prolyl dipeptidyl aminopeptidase. Some of these enzymes can release ACE-inhibitory peptides in fermented dairy beverages (Chen et al., 2015).

Soleymanzadeh et al. (2019) reported the production of ACE-inhibitory peptides in fermented camel milk by *Leuconostoc lactis* and attributed this phenomenon to the proteolytic activity of this bacterium. Chen et al. (2015) evaluated the ACE-inhibitory activity of fermented milk produced by 59 strains of L. *helveticus* and reported that 38 strains exhibited over 50 % ACE inhibition. Additionally, Pihlanto et al. (2010) assessed the ACE-inhibitory potential of 25 different LAB strains used in fermented milk production. Their findings indicated that the ability of LAB to produce ACE inhibitors in milk during fermentation is a strain-dependent characteristic, which, in some cases, is closely related to bacterial growth and proteolytic activity.

Research has shown that ultrasound treatment enhances ACE-inhibitory activity in various dairy and functional beverages. According to Gammoh et al. (2020), applying ultrasound improved the ACE-inhibitory potential of camel milk casein and whey proteins. In another study, Guimaraes et al. (2019) observed that ultrasound treatment boosted the ACE-inhibitory activity of a prebiotic soursop whey beverage, increasing it from 57 % in the untreated sample to 78 % in the ultrasound-exposed sample. Monteiro et al. (2018) also highlighted that utilizing high-intensity ultrasound with elevated energy density led to a notable enhancement in ACE inhibition in chocolate whey beverages.

Studies suggest that ultrasonic pretreatment can alter the molecular conformation of milk proteins, potentially exposing more active groups associated with antihypertensive effects. This structural modification may significantly enhance the ACE-inhibitory activity of proteolysis-derived products (Cui, Yang, Liang, Huang, Lu, Ren, et al., 2020). During ultrasound-assisted processing, the mechanical forces generated by acoustic cavitation promote the disruption and unfolding of protein structures, thereby enhancing their susceptibility to hydrolysis and facilitating the release of bioactive peptides. Additionally, ultrasonication disrupts the aggregation of casein and whey proteins, enhancing their susceptibility to proteolytic enzymes and improving peptide generation (Munir et al., 2020). Ultrasonication, driven by cavitational effects, generates high-energy bubbles that facilitate the dissolution of insoluble casein into the serum phase. This process increases the surface area available for enzymatic hydrolysis, promoting the release of bioactive peptides and protein hydrolysates (Koirala et al., 2021). Additionally, structural modifications in milk proteins, including a reduction in α-helix and β-corner content alongside an increase in β-folding and random coil formations, indicate a loosening of the molecular structure. These alterations enhance protein susceptibility to enzymatic hydrolysis, further improving the production of bioactive compounds (Cui et al., 2020).

### 3.6 Lipase inhibition activity of fermented milk during fermentation

**Fig. 6:**
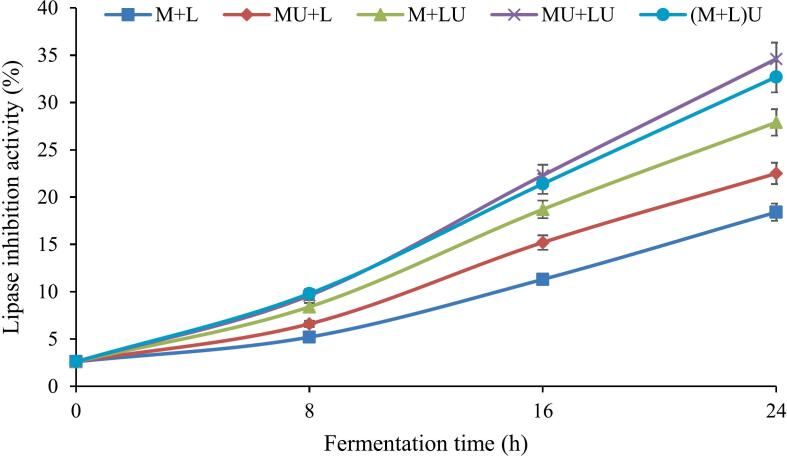
The effect of ultrasound treatment on lipase inhibition activity of fermented milk during fermentation. (MU + L: milk ultrasonicated, M + LU: bacteria ultrasonicated, MU + LU: milk and bacteria ultrasonicated separately, (M + L)U: mixture ultrasonicated after inoculation, control: no ultrasound).

Fig. 6 shows the influence of ultrasonication on lipase inhibition in fermented milk over the fermentation period. According to the results, lipase inhibition significantly (*P* ≤ 0.05) increased in all treatments as fermentation progressed. At the end of fermentation, the M + L treatment (control) exhibited the lowest inhibition level (18.4 %), whereas the MU + LU treatment showed the highest inhibition (34.6 %). Other ultrasound-treated samples, including MU + L, M + LU, and (M + L)U, also displayed greater inhibition than the control, with values of 22.5 %, 27.9 %, and 32.7 %, respectively. Furthermore, at all times, the M + LU treatment exhibited a more pronounced inhibition than MU + L, indicating that ultrasound may exert a more pronounced effect on bacterial cells than on milk components in enhancing lipase inhibition, potentially due to improved cell membrane permeability and enzyme release. From 8 h of fermentation onwards, all ultrasound-treated samples exhibited significantly (P ≤ 0.05) enhanced lipase inhibition compared to the control. Although the MU + LU treatment showed a slightly increased inhibition compared to (M + L)U throughout fermentation, this difference was not significant.

Several studies have demonstrated the ability of various LAB strains to inhibit lipase activity. Gil-Rodríguez and Beresford (2019) investigated the effect of different LAB strains on lipase inhibition in fermented skim milk and reported that L. *helveticus*-fermented milk exhibited the highest levels of lipase inhibition. Similarly, Azadikhah et al. (2025) assessed the impact of 20 distinct LAB strains used in probiotic fermented milk production and found that most fermented samples displayed higher lipase inhibitory activity compared to the control. In a related study, Gil-Rodríguez and Beresford (2021) examined the lipase inhibitory potential of fermented milk produced using 71 LAB strains from the genera *Lactobacillus*, *Lactococcus*, *Leuconostoc*, and *Pediococcus*. Their findings revealed that the most fermented samples exhibited greater lipase inhibition than their non-fermented counterparts. They further emphasized that the development of lipase inhibitory activity during milk fermentation is strain-dependent, as different strains within the same species exhibited varying levels of inhibition.

Different strains of LAB exhibit varying levels of enzyme inhibition due to differences in their proteolytic enzyme profiles and transport systems (Kinariwala et al., 2020). During milk fermentation, LAB promote protein hydrolysis through their proteolytic activity, leading to the release of free amino groups, polypeptides, oligopeptides, and smaller peptides (Aguilar-Toalá et al., 2017). Studies have shown that LAB cells and their extracts can inhibit pancreatic lipase. However, this inhibitory effect may also stem from bioactive compounds generated during fermentation, such as organic acids and bioactive peptides (Kojta et al., 2020).

The characteristics of peptides play a crucial role in their ability to bind to the enzyme's active site and inhibit its activity. Bioactive peptide extracts can effectively reduce lipase activity by blocking key active sites responsible for triglyceride hydrolysis. Additionally, the presence of amino acid residues with hydrophobic properties in these peptides plays a crucial role in inhibiting pancreatic lipase, highlighting hydrophobicity as a key characteristic of potent lipase inhibitors. Moreover, the molecular weight of peptides significantly impacts their inhibitory potential, with lower molecular weight peptides demonstrating greater enzyme inhibition efficiency (Azadikhah et al., 2025).

Mudgil et al. (2022) reported that ultrasound application enhanced lipase inhibition in bovine milk compared to the control. This improvement is likely associated with ultrasound-induced modifications of milk proteins, which may facilitate the release of bioactive peptides. However, the exact mechanisms remain to be fully elucidated. Furthermore, ultrasonication enhances the solubility and bioavailability of peptides, thereby strengthening their interactions with pancreatic lipase. Mechanistically, this enhancement can be attributed to ultrasound-induced cavitation, which disrupts the tertiary and quaternary structures of milk proteins and increases their susceptibility to enzymatic hydrolysis. These structural modifications may facilitate the release of bioactive peptides with inhibitory activity against lipase. Nevertheless, the precise identity, structural characteristics, and interaction mechanisms of these peptides with the enzyme remain unclear and require further elucidation. Also, additional studies involving peptide sequencing and molecular docking are needed to clarify the specific mechanisms underlying lipase inhibition. Moreover, the combined application of ultrasound and fermentation appears to synergistically enhance the production of bioactive metabolites, as evidenced by the higher lipase inhibition observed in the MU + LU treatment.

## 4 Conclusions

Ultrasound treatment significantly enhanced the microbial viability and bioactive properties of L. *helveticus*-fermented milk. The synergistic effect observed when ultrasound was applied to milk and bacteria (MU + LU) led to the most pronounced improvements in bacterial growth, acidification, antioxidant capacity, and enzyme inhibitory activities. These findings suggest that ultrasound can effectively accelerate fermentation kinetics and enhance the functional attributes of fermented dairy products, making them more nutritionally and therapeutically beneficial. The increased production of bioactive compounds, including antioxidant peptides and enzymes with anti-diabetic and anti-hypertensive properties, further underscores the potential of ultrasound as a non-thermal processing technology for developing functional foods. Given its advantages over conventional methods, ultrasound offers a promising and sustainable approach to improving the quality and health-promoting properties of fermented dairy products. Future research could explore the optimal ultrasound parameters and their effect on other microorganisms and dairy matrices, further expanding the application of this technology in the food industry.

## CRediT authorship contribution statement

**Aliakbar Gholamhosseinpour:** Writing – original draft, Software, Formal analysis, Data curation, Conceptualization. **Seyed Mohammad Bagher Hashemi:** Writing – review & editing, Visualization, Supervision. **Hamideh Khosravi Mazydi:** Investigation, Data curation.

## Declaration of competing interest

The authors declare that they have no known competing financial interests or personal relationships that could have appeared to influence the work reported in this paper.

## Data Availability

Data will be made available on request.
